# Rosiglitazone Prevents High Glucose-Induced Vascular Endothelial Growth Factor and Collagen IV Expression in Cultured Mesangial Cells

**DOI:** 10.1155/2009/910783

**Published:** 2009-07-07

**Authors:** Catharine Whiteside, Hong Wang, Ling Xia, Snezana Munk, Howard J. Goldberg, I. George Fantus

**Affiliations:** ^1^Department of Medicine, University Health Network, Toronto, ON, Canada M5S 1A8; ^2^University of Toronto, Toronto, ON, Canada M5S 1A8; ^3^Department of Medicine, Mount Sinai Hospital, University of Toronto, Toronto, ON, Canada M5S 1A8

## Abstract

Peroxisome proliferator-activated receptor (PPAR*γ*), a ligand-dependent transcription factor, negatively modulates high glucose effects. We postulated that rosiglitazone (RSG), an activator of PPAR*γ* prevents the upregulation of vascular endothelial growth factor (VEGF) and collagen IV by mesangial cells exposed to high glucose. Primary cultured rat mesangial cells were growth-arrested in 5.6 mM (NG) or 25 mM D-glucose (HG) for up to 48 hours. In HG, PPAR*γ* mRNA and protein were reduced within 3 h, and enhanced ROS generation, expression of p22^phox^, VEGF and collagen IV, and PKC-*ζ* membrane association were prevented by RSG. In NG, inhibition of PPAR*γ* caused ROS generation and VEGF expression that were unchanged by RSG. Reduced AMP-activated protein kinase (AMPK) phosphorylation in HG was unchanged with RSG, and VEGF expression was unaffected by AMPK inhibition. Hence, PPAR*γ* is a negative modulator of HG-induced signaling that acts through PKC-*ζ* but not AMPK and regulates VEGF and collagen IV expression by mesangial cells.

## 1. Introduction

The hallmark of progressive diabetic glomerulosclerosis is the accumulation of excessive extracellular matrix protein (ECM), mainly collagen IV, in the glomerular interstitium [1, 2]. This results in large part from the transformation of quiescent mesangial cells to a dedifferentiated myofibroblast phenotype as a result of the direct effects of high glucose and the response to autocrine and paracrine growth factors including vascular endothelial growth factor (VEGF) and transforming growth factor (TGF)-*β* [3, 4]. We and others have demonstrated that early mesangial cell responses to high glucose include the generation of reactive oxygen species (ROS) from NADPH oxidase, a necessary signaling factor in the stimulation of VEGF and collagen IV expression [3, 5]. 

 Recent studies have suggested that peroxisome proliferator-activated receptor-*γ * (PPAR*γ*) synthetic agonist thiazolidinediones, such as rosiglitazone, may prevent or attenuate diabetic nephropathy in animal models [6, 7]. PPAR*γ* is a member of the nuclear receptor superfamily of ligand-activated transcription factors. Upon ligand binding, PPAR*γ* forms a heterodimer with the retinoic X receptor. This complex then binds to PPAR response elements (PPREs) within the promoter region of target genes [8]. PPAR*γ* agonists have been shown to play an important role in regulating adipocyte differentiation, lipid and glucose metabolism, and inflammation [9]. Asano et al. [10] reported that rat mesangial cells express PPAR*γ* localized in the nucleus, and that troglitazone (an agonist of PPAR*γ*) prevents cellular dedifferentiation as detected by reduced expression of *α*-smooth muscle actin expression. PPAR*γ* agonists inhibit TGF-*β*_1_ [11] and Ang II [12] stimulation of vascular smooth muscle cells, and mesangial cell proliferation and fibronectin synthesis in response to VEGF [13] and TGF-*β*_1_ [14], respectively. 

We have demonstrated that in response to high glucose, mesangial cells rapidly express and secrete VEGF that is dependent on the activation of both PKC-*β* and PKC-*ζ*, and the generation of reactive oxygen species (ROSs) [15]. Yang et al. reported [16] that hyperglycemic Zucker rats develop increased circulating VEGF and that the PPAR*γ* agonist pioglitazone normalized serum glucose and VEGF levels. Onozaki et al. [13] showed that during exposure to a rapid change in ambient glucose concentration, mesangial cell proliferation dependent on VEGF expression was inhibited by a thiazolidinedione. The cellular signaling mechanisms that connect the effects of high glucose to altered mesangial cell PPAR*γ* expression and function and consequent outcomes relevant to progressive glomerulosclerosis are unknown. 

In this study, we postulated that rosiglitazone would reverse the effects of high glucose essential for the early responses of mesangial cells associated with myofibroblast transformation including ROS generation, VEGF and collagen IV expression. To identify the role of PPAR*γ* in mesangial cells, we tracked its expression and the effects of rosiglitazone during exposure to high glucose. The actions of rosiglitazone on high glucose-stimulated ROS generation via NADPH oxidase and the expression of VEGF and collagen IV were observed. These effects were confirmed by similar findings with two other PPAR*γ* agonists, Ciglitazone and Troglitazone. Supporting these observations, a specific inhibitor of PPAR*γ*, GW9662, on mesangial cell expression of VEGF in normal glucose and high glucose had opposite effects. To determine whether AMP-activated protein kinase (AMPK), reported to be activated by thiazolidinediones, is involved in this mesangial cell PPAR*γ* pathway, the phosphorylation of AMPK was analyzed in the presence of Compound C, a specific antagonist of AMPK [17], with and without rosiglitazone. Our data support a major role for downregulation of PPAR*γ* during the early response of mesangial cells to high glucose and reversal with rosiglitazone. 

## 2. Materials and Methods

### 2.1. Materials

Dulbecco's modified Eagle medium (DMEM) and fetal bovine serum (FBS) were purchased from Invitrogen Corporation (Burlington, Ont, Canada). 5-(and-6)-chlormethyl-2′,7′-dichlorodihydrofluorescein diacetate (CM-H_2_DCFDA) was obtained from Molecular Probes Inc. (Eugene, Ore, USA). Rabbit Polyclonal antibodies against p22^phox^ and VEGF, and monoclonal antibodies against PPAR*γ* were obtained from Santa Cruz Biotechnology, Inc. (Santa Cruz, Calif, USA). Monoclonal antibody against *β*-actin was purchased from Sigma-Aldrich (St, Louis, Mo, USA). Rabbit polyclonal antibody against type IV collagen *α* was purchased from Rockland Immunochemicals (Gilbertsville, Pa, USA). The rabbit polyclonal antibodies against phospho- and total-AMPK alpha were purchased from Cell Signaling Technology, Inc. (Danvers, Mass, USA). The selective ATP-competitive inhibitor of AMPK, Compound C, and Ciglitazone were purchased from Calbiochem (Gibbstown, NJ, USA). Rosiglitazone and Troglitazone and GW9662 were purchased from Cayman Chemical (Ann Arbor, Mich, USA).

### 2.2. Cell Culture

Primary rat glomerular mesangial cells were isolated from Sprague-Dawley rat kidney cortex and cultured as previously described [18, 19]. The cells were cultured in DMEM containing 17% FBS, then routinely growth-arrested in 0.5% FBS for 48 hours in either normal D-glucose 5.6 mM or high D-glucose 25 mM, or 5.6 mM D-glucose + 24.4 mM L-glucose for up to 48 hours. In some experiments, cells were incubated with 10 *μ*M rosiglitazone, 10 *μ*M Ciglitazone, 10 *μ*M Troglitazone, and/or pretreated with 10 *μ*M GW9662 (a PPAR*γ* antagonist). AMPK activity was inhibited by pretreatment for 48 hours with 50 uM Compound C, a cell-permeable, selective ATP-competitive kinase inhibitor of AMPK [17, 20, 21]. The glitazone compounds were first dissolved in DMSO to create a 25.2 mmol/uL stock solution stored at −20°C and then dissolved in DMEM to produce a final concentration of 10 uM in the cell culture medium.

### 2.3. Western Immunoblotting

Western immunoblots were performed with primary antibodies against PPAR*γ*, p22^phox^, VEGF, AMPK, *β*-actin in total cell lysates, or PKC-*ζ*, -*β*_1_ in total cell lysates and cellular membrane fractions as we previously described [5, 15].

### 2.4. Quantitative Real-Time Polymerase Chain Reaction

Total cellular RNA was extracted from mesangial cells using an RNeasy kit (Qiagen, Valencia, Calif, USA). After the RNA was reverse transcribed, real-time PCR was performed with the following primers as described previously [15]. The primers for VEGF were (sense), 5′-GATGAGATAGAGTATATCTTCAAGCCGT-3′, and (antisense), 5′-TCTATCTTTCTTTGGTCTGCATTCAC-3′ (GenBank: NM_031836). The primers for p22^phox^ were (sense), 5′-TCCTCCACTTACTGCTGTCCGT-3′, and (antisense), 5′-TCAATGGGAGTCCACTGCTCAC -3′ (Genbank: MIM_131550).The primers for PPAR*γ* were (sense) 5′-CCAGAGTCTGCTGATCTGCGA-3′, and (antisense), 5′-GCCACCTCTTTGCTCTGCTC-3′ (Genbank: MIM_131550). The primers for *β*-actin were (sense) 5′-AGGCCCCTCTGAACCCTAAG-3′, and (antisense), 5′-CAACACAGCCTGGATGGCTAC-3′ (Genbank: NM_031144).

### 2.5. Measurement of PPAR*γ*Promoter Activity

To assess PPAR*γ* function, mesangial cells were transiently transfected with a luciferase reporter gene containing three PPAR*γ* response elements and a thymidine kinase promoter [22] obtained from Addgene (Cambridge, Mass, USA). Cells were plated in 24 well plates and transfected with Fugen*e* 6 (Roche, Indianapolis, Ind, USA) according to the manufacturer's instructions. For measurement of luciferase activity, the transfected mesangial cells were growth-arrested in 0.5% FBS in 5.6 mM or 25 mM D-glucose for up to 48 hours. In some experiments, 10 uM rosiglitazone or/and 10 *μ*M GW9662 was added to the medium for 48 hours. The mesangial cells were then lysed on ice in a buffer containing glycylglycine 25 mM, pH 8, MgSO_4_ 15 mM, EGTA 4 mM, 1% Triton X-100. Luciferase activity was detected in 50 uL of cell extract plus 100 uL of reaction buffer (glycylglycine 25 mM, pH 8, KH_2_PO_4_ 15 mM, EGTA 4 mM, ATP 2 mM, MgSO_4_ 15 mM, and CoA 0.1 mM) over 20 seconds in a plate reading luminometer. The results were normalized to total cell protein.

### 2.6. Confocal Imaging

To analyze collagen IV protein content, cells were cultured on glass coverslips and incubated with polyclonal antibody against collagen IV. The primary antibodies were detected using FITC-conjugated goat anti-rabbit IgG (Jackson Immunoresearch Laboratories, Inc; West Grove, Pa, USA). Immunofluorescence was observed by confocal imaging and fluorescence intensity per cell was analyzed as previously described [5, 15].

To analyze hydrogen peroxide content, mesangial cells were cultured on glass coverslips and incubated in the dark with 1 *μ*M of CM-H_2_DCFDA for 25 minutes at 37°C. Intracellular ROS production was detected by confocal laser scanning microscopy. Fluorescence intensity per cell was analyzed by Scion Image software (Scion Corporation, Frederick, Md, USA) as previously described [5, 15].

### 2.7. Statistical Analyses

 All results are expressed as mean ± SEM. Statistical analyses were performed using Instat 2.01 (Graph Pad, Sacramento, Calif, USA). Unpaired Student *t* tests were used to compare the means of two groups. One-way analysis of variance (ANOVA) was performed to compare the means of three groups or more, then the Tukey-Kramer multiple comparison test was applied for post test analysis. *P* < .05 was considered to be statistically significant.

## 3. Results

### 3.1. Effects of High Glucose and Rosiglitazone on PPAR*γ* Expression

**Figure 1:**
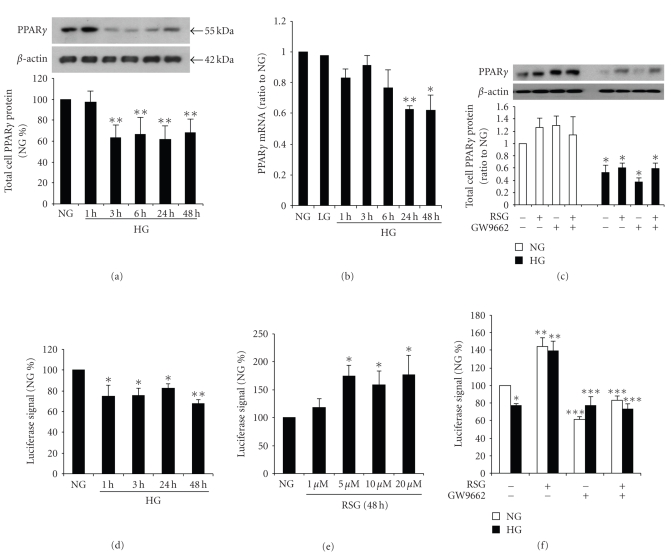
*Effect of high glucose on PPAR* *γ* *expression. * Rat glomerular mesangial cells were cultured in 5.6 mM (NG) or 25 mM D-glucose (HG), or 5.6 mM D-glucose + 24.4 mM L-glucose (LG) for up to 48 hours. (a) PPAR*γ* was detected by immunobloting in total cell lysates, using *β*-actin as the loading controls. The graphs represent PPAR*γ* protein levels relative to NG. (b) PPAR*γ* mRNA levels were determined by real-time RT-PCR. (*n* = 4–6, *P < .05 versus NG; **P < .01 versus NG). (c) Preincubated with 10 *μ*M rosiglitazone (RSG) or/and 10 *μ*M GW9662, the protein expression of PPAR*γ* was not affected by RSG or GW6992 in HG (*n* = 5, *P < .05 versus NG). Mesangial cells were transiently transfected with a luciferase reporter gene containing three PPAR response elements and then cultured in the above conditions. (d) Luciferase reporter activity was reduced in HG (*n* = 5, *P < .05 versus NG, **P < .01 versus NG). (e) In NG, PPAR*γ *promoter activity increased in dose response to RSG (*n* = 4, *P < .01 versus NG). (f) GW6992 blocked the effect of RSG (*n* = 4–6, *P < .01 versus NG alone, **P < .05 versus NG or HG alone, ***P < .05 versus NG or HG with RSG).

Mesangial cell expression of PPAR*γ* in high glucose was analyzed by incubating cells with 5.6 mM (normal glucose) or 25 mM (high glucose) D-glucose, or 5.6 mM D-glucose + 24.4 mM L-glucose for up to 48 hours, with and without rosiglitazone. L-Glucose exposure for 48 hours had no effect on PPAR*γ* mRNA expression. Figure 1(a) shows that in high glucose, PPAR*γ* protein expression was reduced by 3 hours and sustained up to 48 hours as demonstrated by Western immunoblot. PPAR*γ* mRNA levels were significantly reduced by 24 hours continuing up to 48 hours Figure 1(b). We also demonstrated that neither rosiglitazone nor GW6992 (an antagonist of PPAR*γ*), altered PPAR*γ * protein levels in high glucose [17, 23] Figure 1(c).

To investigate whether PPAR*γ* activation of transcription is reduced in high glucose and if rosiglitazone alters this response, mesangial cells were transiently transfected with a PPAR*γ*-luciferase reporter gene as described by others [24, 25]. Decreased luciferase activity was found at 1 to 48 hours of exposure to high glucose as shown in Figure 1(d). These data suggest that downregulation of PPAR*γ* expression in high glucose reduces the functional effect of PPAR*γ* on promoter activity. To determine whether rosiglitazone modulates PPAR*γ* stimulation of promoter activity, mesangial cells were pre-incubated with 1 to 20 uM rosiglitazone and promoter activity was measured. A maximum effect on luciferase activity was observed in normal glucose in the cells incubated with 5 uM rosiglitazone Figure 1(e), and in high glucose, 10 uM rosiglitazone stimulated luciferase to a similar maximum Figure 1(f). We then found that inhibition of PPAR*γ* receptor activity with GW6992 prevented the rosiglitazone-stimulated PPAR-*γ* responsive promoter activity both in normal glucose and high glucose Figure 1(f).

### 3.2. ROS Generation Regulated by PPAR*γ*

**Figure 2:**
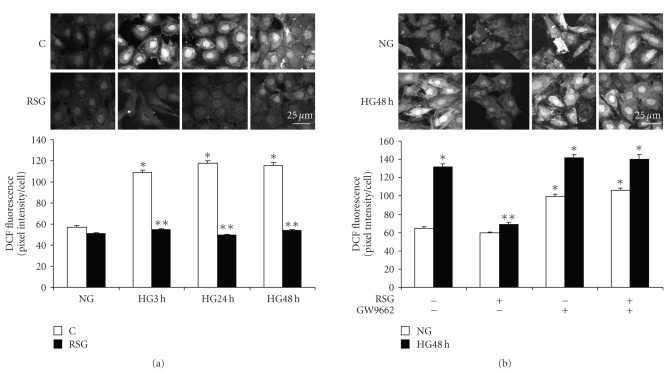
*Effect of rosiglitazone on reactive oxygen species generation. * Mesangial cells were exposed to 5.6 mM (NG) or 25 mM (HG) D-glucose for up to 48 hours, and pretreated with 10 *μ*M rosiglitazone (RSG) or GW9662 for 1 hour. Reactive oxygen species (ROS) were detected in DCF-loaded cells by confocal fluorescence imaging in 3 or 4 separate experiments for each condition. (a) ROS generation was inhibited by RSG (*n* = 147–218 cells, *P < .001 versus NG, **P < .001 versus HG 3 hours, HG 24 hours, or HG 48 hours). (b) ROS generation in NG was stimulated with GW9662 (*n* = 130–189 cells, *P < .001 versus NG, **P < .001 versus HG 48 hours without RSG or GW9662). Magnification bar = 25 *μ*M.

 In high glucose, ROS generation appeared within 1 to 3 hours in DCF-loaded mesangial cells (Figure 2), as previously reported [5, 15]. We observed that 1 hour pretreatment with rosiglitazone abolished ROS generation during 3 to 48 hours of exposure to high glucose Figure 2(a). This effect of rosiglitazone was blocked by preincubation with GW9662 Figure 2(b). GW9662 alone caused generation of ROS in normal glucose. 

**Figure 3:**
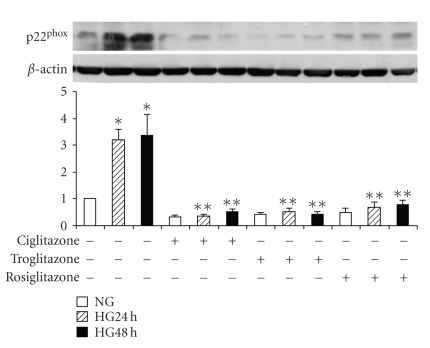
*NADPH oxidase subunit * p22^phox^ *expression. * Mesangial cells were exposed to 5.6 mM (NG) or 25 mM (HG) D-glucose for 24 hours or 48 hours, or pretreated with 10 *μ*M rosiglitazone (RSG), or 10 *μ*M ciglitazone or 10 *μ*M troglitazone) and p22^phox^ was detected by immunoblotting. Pretreatment with three different PPAR*γ* agonists blocked HG-induced p22^phox^ expression (*n* = 5, *P < .05 versus NG, **P < .05 versus HG 24 hours or HG 48 hours).

We have reported that the NADPH oxidase subunit, p22^phox^ is upregulated in high glucose in mesangial cells [15]. To determine whether PPAR*γ* activation modifies high glucose-induced p22^phox^ expression, mesangial cells were exposed to 5.6 mM or 25 mM D-glucose for up to 48 hours. First, the cells were preincubated with 10 *μ*M rosiglitazone alone. As displayed in Figure 3(a), p22^phox^ protein levels in high glucose were increased as expected at 24 hours and 48 hours. Rosiglitazone not only prevented the effect of high glucose on p22^phox^ protein but also reduced the ambient level of this protein in normal glucose. Further, cells were tested with three different PPAR*γ* activators that all blocked high glucose-induced p22^phox^ protein expression as shown in Figure 3(b).

### 3.3. Rosiglitazone Prevents High Glucose-Induced VEGF and Collagen IV Expression in Mesangial Cells

**Figure 4:**
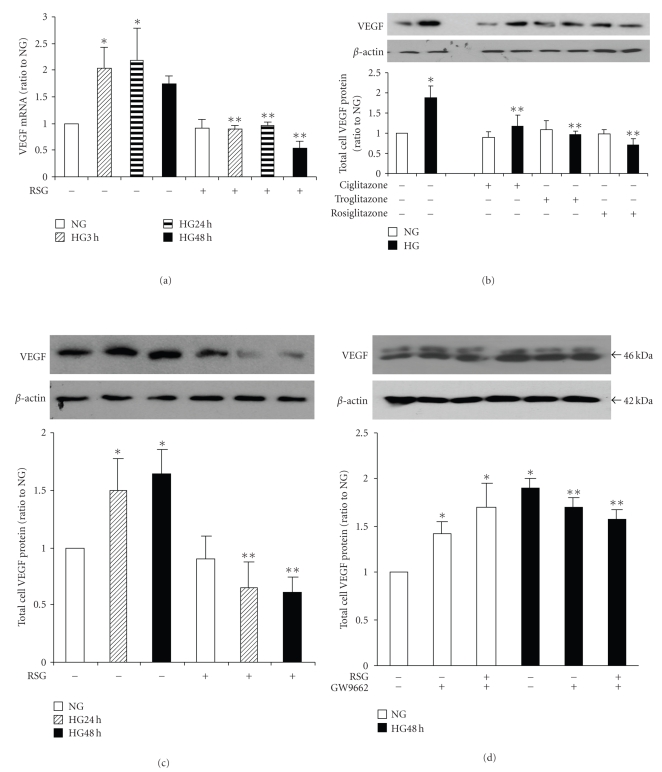
*Rosiglitazone prevented high glucose-induced VEGF expression*. Mesangial cells were placed in 5.6 mM (NG) or 25 mM (HG) D-glucose for the indicated times or pretreated with PPAR*γ* agonists (10 *μ*M Ciglitazone, or 10 *μ*M Troglitazone, or 10 *μ*M RGS) for 1 hour. (a) VEGF mRNA levels were detected by real-time PCR (*n* = 4, *P < .05 versus NG, **P < .01 versus HG). (b) Pretreatment with three different PPAR*γ* agonists similarly blocked HG-induced VEGF expression at 48 hours as detected by immunobloting (*n* = 4, *P < .01 versus NG, **P < .01 versus HG). (c) Rosiglitazone alone prevented HG-induced VEGF expression at both 24 hours and 48 hours as detected by immunoblotting in total cell lysates using *β*-actin as the loading control. The graphs represent VEGF protein levels relative to NG (*n* = 5, *P < .01 versus NG, **P < .01 versus HG). (d) GW9662 reverses the effect of RSG, (*n* = 5, *P < .01 versus NG without RSG or GW9662, **P < .001 versus NG without RSG or GW9662).

To test the effect of PPAR*γ* activation and inhibition on VEGF expression, mesangial cells were growth-arrested and exposed to 5.6 mM or 25 mM D-glucose for up to 48 hours, with or without rosiglitazone or GW9662. The increase in VEGF mRNA levels was prevented by pretreatment with rosiglitazone Figure 4(a). Both Ciglitazone and Troglitazone also blocked high glucose-stimulated VEGF expression at 48 hours as detected by Western immunoblot Figure 4(b). Then, enhanced VEGF protein expression in high glucose was inhibited by pretreatment with 10 uM rosiglitazone at both 24 hours and 48 hours Figure 4(c) as detected by Western immunoblot. The inhibition of PPAR*γ* with GW9662 alone in normal glucose caused a significant increase in VEGF expression. Furthermore, this inhibitor reversed the effect of rosiglitazone on VEGF expression in mesangial cells in high glucose Figure 4(d). 

**Figure 5:**
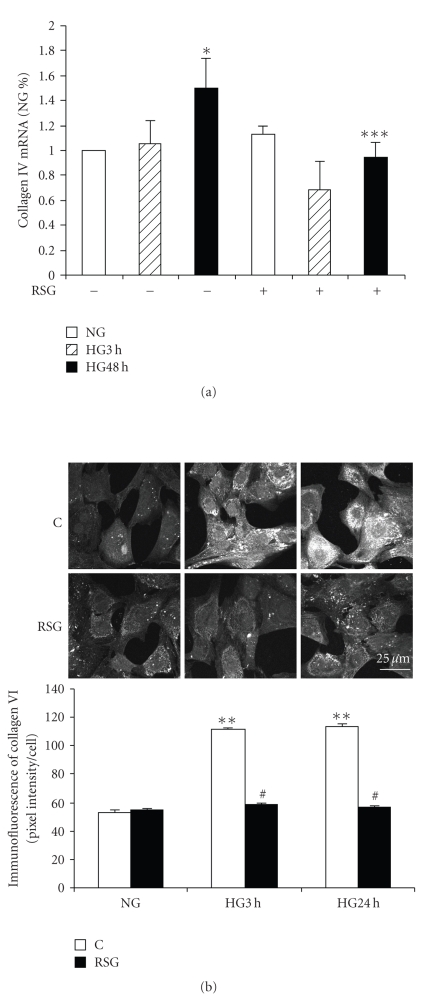
*Rosiglitazone prevented high glucose-induced collagen IV expression*. Mesangial cells were exposed to 5.6 mM (NG) or 25 mM (HG) for up to 48 hours pretreated with 10 *μ*M rosiglitazone (RSG) for 1 hour. (a) The expression of collagen IV (*α*1) mRNA was detected by real-time RT-PCR (*n* = 4, *P < .05 versus NG). (b) Representative confocal microscopic images show collagen IV staining without (C) or with RSG. Immunofluorescence intensity per cell was analyzed (*n* = 76–136 cells, **P < .01 versus NG). Magnification bar = 25 *μ*M.

To determine the effect of rosiglitazone on collagen IV expression, we first analyzed the effect of this PPAR*γ* activator on high glucose-induced mRNA expression. A significant increase in collagen IV mRNA was observed after 48 hours, but not 3 hours, in high glucose and this response was inhibited by rosiglitazone Figure 5(a). Intracellular collagen IV protein expression was assessed by immunofluorescence imaging. The increase in collagen IV protein at 3 and 24 hours was prevented by rosiglitazone pretreatment Figure 5(b).

### 3.4. Effect of Rosiglitazone on PKC-*β*_1_ and -*ζ* Membrane Translocation

**Figure 6:**
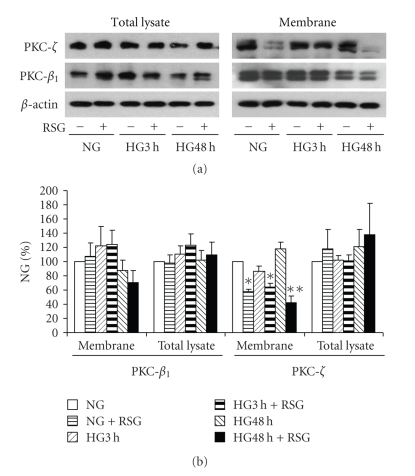
*PKC-*β*_1_ and PKC-*ζ* response to high glucose and rosiglitazone*. Mesangial cells were exposed to 5.6 mM (NG) or 25 mM (HG) for 3 hours or 48 hours pretreated with 10 *μ*M rosiglitazone (RSG) for 1 hour. PKC-*β*_1_ and PKC-*ζ* were detected in total cell lysates or membrane fractions with immunoblotting. (a) Representative immunoblots of PKC-*β*_1_ and PKC-*ζ*. (b) Quantitative analysis of the immunoblots (*n* = 5, *P < .05 versus NG without RSG, **P < .01 versus HG without RSG).

Since our previous studies indicated a cause-and-effect relationship among the activation of PKC-*β*_1_ and PKC-*ζ*, ROS generation and both VEGF and collagen IV expression [5, 15], we wished to test whether rosiglitazone could affect these two key PKC isozymes that are relevant to the pathogenesis of diabetic glomerulophathy. As illustrated in Figure 6, in the presence of rosiglitazone, a reduction in membrane-association of PKC-*ζ*, but not PKC-*β*_1_, was observed in both normal and high glucose. Total recoveries of both PKC isozymes in total cell lysate were unchanged in high glucose or in the presence of rosiglitazone.

### 3.5. Rosiglitazone and the AMPK Pathway

**Figure 7:**
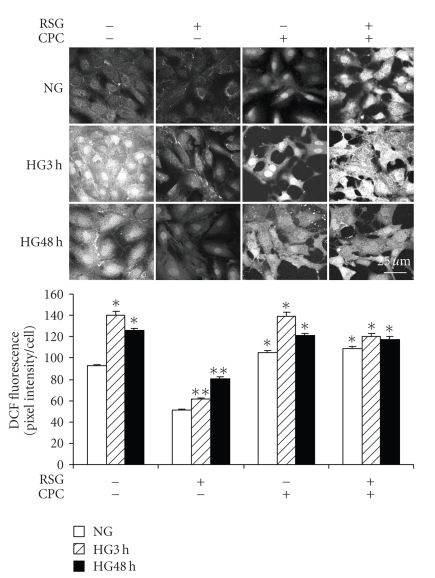
*Compound C reversed rosiglitazone prevented high glucose-induced reactive oxygen species generation*. Mesangial cells were exposed to 5.6 mM (NG) or 25 mM (HG) for 3 hours or 48 hours, and then pretreated with 10 *μ*M rosiglitazone (RSG) or 50 *μ*M compound C (CPC), or combination of both, for 1 hour. Reactive oxygen species (ROS) generation was measured in DCF-loaded cells using confocal fluorescence imaging. RSG prevented high glucose stimulated ROS production that was abolished by coincubation with CPC (*n* = 145–204 cells, *P < .05 versus NG in the absence of RSG and/or CPC, **P < .01 versus HG in the absence of RSG and/or CPC). Magnification bar = 25 *μ*M.

 It has been reported that rosiglitazone reduces ROS production by NADPH oxidase independent of PPAR*γ* activity and that this effect may involve the AMPK pathway [17]. Thus, we determined whether inhibition of AMPK with Compound C would reverse the inhibition of ROS generation in response to HG observed during rosiglitazone treatment. Mesangial cells were growth-arrested and exposed to 5.6 mM or 25 mM D-glucose for up to 3 hours or 48 hours, with or without 1 hour pretreatment with 10 *μ*M rosiglitazone or 50 *μ*M Compound C, or both. In Figure 7, the effect of rosiglitazone on high glucose-induced ROS generation was reversed by coincubation with Compound C. Of note was that Compound C alone caused ROS generation in normal glucose at 48 hours. The enhanced generation of ROS by mesangial cells observed in high glucose at 3 and 48 hours was not affected by Compound C. However, in the presence of Compound C, the effect of rosiglitazone on ROS generation in high glucose was reversed in keeping with a possible AMPK-independent effect of Compound C on ROS generation.

**Figure 8:**
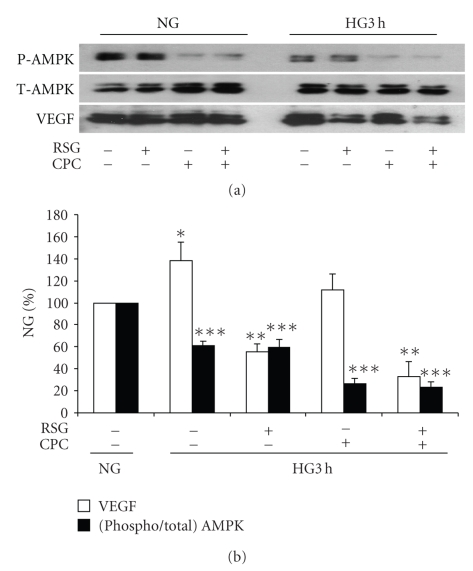
*Effects of Compound C on AMP kinase activity and VEGF in high glucose*. Mesangial cells were exposed to 5.6 mM (NG) or 25 mM (HG) for 3 hours, and then pretreated with 10 *μ*M rosiglitazone (RSG) or 50 *μ*M compound C (CPC), or combination of both, for 1 hour. Total AMP kinase (T-AMPK), phosphorylated AMPK (P-AMPK) and VEGF were detected in the same total cell lysate samples with immunoblotting. CPC inhibited phosphorylation of AMPK in NG and HG, but had no effect on the inhibition of VEGF expression in HG. RSG had no effect on P-AMPK. (*n* = 3, *P < .05 versus VEGF in NG, **P < .05 versus VEGF in HG 3 hours without RSG or CPC, ***P < .05 versus P-AMPK in HG 3 hours without RSG or CPC).

The phosphorylation of AMPK in normal and high glucose at 3 hours was inhibited with Compound C as illustrated in Figure 8. In view of the generation of ROS during exposure to Compound C, these experiments were limited to 3 hours. VEGF protein expression in high glucose was also analyzed in the same protein samples. Rosiglitazone had no effect on AMPK phosphorylation in either normal or high glucose. While Compound C, as expected, inhibited AMPK phosphorylation in both normal and high glucose, it had no effect on VEGF expression in normal or high glucose. Furthermore, the inhibitory effect of rosiglitazone on enhanced VEGF expression at 3 hours in high glucose was not significantly affected by Compound C Figure 8(b).

## 4. Discussion

In this study, we identified that within the first 6 hours of exposure to high glucose, mesangial cell PPAR*γ* is downregulated and that rosiglitazone prevents the effects of high glucose on NADPH oxidase-dependent ROS generation, VEGF and collagen IV expression. The analysis of PPAR*γ* protein levels indicated a reduction as early as 3 hours following high glucose exposure, although significant reduction in PPAR*γ* mRNA levels was not detected until 6 hours, suggesting that high glucose may differentially alter translation and transcription. Differential regulation of PPAR*γ* protein and mRNA levels in high glucose could also be due to enhanced protein degradation following activation as demonstrated by Hauser et al. [26]. We demonstrated the dose response of the PPAR*γ*-stimulated promoter activity in response to increasing concentrations of rosiglitazone. Two other PPAR*γ* agonists, Ciglitazone and Troglitazone prevented high glucose-induced p22^phox^ and VEGF expression. Our data support the conclusion that rosiglitazone prevents the effects of high glucose on mesangial cell signaling and gene expression through PPAR*γ*.

We also found that rosiglitazone prevented high glucose-induced upregulation of NADPH oxidase subunit, p22^phox^ expression, correlating with the effect of rosiglitazone in attenuating ROS generation in response to high glucose. Further, an antagonist of the PPAR*γ* receptor, GW9662, caused ROS generation in normal glucose and prevented the effects of rosiglitazone. These data also support the conclusion that the effects of rosiglitazone in mesangial cells are likely mediated through PPAR*γ*. Our findings agree with those recently reported by Henderson et al. [27] and Tyagi et al. [28] who found that a PPAR*γ* agonist (Ciglitazone) prevented the ROS generation that was associated with an upregulation of the NADPH oxidase subunit (Nox4) in response to pressure overload and homocysteine in myocardium and in endothelial cells, respectively. The combination of PPAR*α* and *γ* activators also inhibits angiotensin II-induced ROS production by NADPH oxidase in hypertensive rats [29]. Hwang et al. [30] recently reported that rosiglitazone reduces vascular oxidative stress and NADPH oxidase subunit expression in diabetic mice. We have shown that sustained production of ROS in mesangial cells in high glucose may be due to the upregulation of NADPH oxidase subunits, p47^phox^ and p22^phox^ via a PKC-dependent mechanism [5, 31]. The present study extends our findings to include a role for PPAR*γ* in the negative regulation of p22^phox^ expression in mesangial cells in the normal state as well as reversal of the increase induced by high glucose.

Recently, we published that in high glucose the upregulation of VEGF expression by mesangial cells is dependent on ROS generation by NADPH oxidase [15]. Evidence is increasing for an important functional relationship between PPAR and VEGF. Xin et al. [32] reported that the activation of PPAR*γ* with Ciglitazone in human umbilical vein endothelial cells reduced VEGF receptor 1 (Flt1) and 2 (Flk/KDR) expression. Further, Meissner et al. [33] demonstrated that PPAR*α* activators inhibit VEGF receptor 2 expression via inhibition of Sp1-dependent DNA binding and trans-activation. In endothelial cells, PPAR*γ* activators inhibited VEGF-induced AKT phosphorylation and consequent endothelial cell migration [34]. Our present data are consistent with these reports and show that rosiglitazone abolished high glucose-stimulated VEGF expression in mesangial cells. 

Our findings also illustrate that rosiglitazone prevented collagen IV expression by mesangial cells in high glucose. These results are in keeping with a number of studies relating PPAR activation to the prevention of extracellular matrix production relevant to kidney disease. Accelerated nephropathy is observed in diabetic PPAR*α*-knockout mice due to collagen IV deposition [35]. The PPAR*α* agonist fenofibrate prevents diabetic nephropathy in db/db mice [36]. In human kidney fibroblasts, the PPAR*γ* agonist, pioglitazone, reduces extracellular matrix production [37]. In mesangial cells, the PPAR*γ* agonist thiazolidinediones inhibit TGF-*β*_1_-induced fibronectin expression and ameliorate diabetic nephropathy [38, 39]. In renal tubular cells, pioglitazone activation of PPAR*γ* exerts antifibrotic effects in the setting of high glucose [40]. These findings do not exclude the possibility that PPAR*γ* activation in vivo may also prevent the accumulation of collagen IV through suppression of plasminogen activator inhibitor-1 [41]. 

To identify the mechanism(s) whereby PPAR*γ* regulates mesangial cell signaling and gene expression, we examined the effect of rosiglitazone on PKC-*β*_1_ and -*ζ* membrane-association patterns and on AMPK phosphorylation. The membrane-association of PKC-*ζ* was inhibited by rosiglitazone, whereas PKC-*β*_1_ was unaffected. We previously demonstrated the cause-and-effect relationship between the activation of mesangial cell PKC-*ζ* in high glucose and subsequent generation of ROS, VEGF, and collagen IV expression in response to high glucose [5, 15]. Therefore, the observation that a PPAR*γ* activator appears to inhibit PKC-*ζ* membrane association links PPAR*γ* to this specific PKC isozyme pathway. By contrast, rosiglitazone had no effect on AMPK phosphorylation, suggesting that PPAR*γ* in mesangial cells may not signal through this pathway. These results differ from a recent report in which rosiglitazone was observed to reduce glucose-induced oxidative stress mediated by NADPH oxidase via an AMPK-dependent mechanism in endothelial cells [17, 42]. In human neutrophils, AMPK activation inhibits ROS generation by NADPH oxidase [23]. We observed that in high glucose, mesangial cell AMPK phosphorylation was reduced. Taken together with the observation that inhibition of AMPK with Compound C was associated with ROS generation, it is possible that AMPK regulates ROS generation in mesangial cells, but separately from the action of PPAR*γ*. It appears that the signaling response of AMPK in high glucose may vary depending on the cell phenotype. 

The precise mechanism whereby PPAR*γ* inhibits high glucose-stimulated VEGF, ROS formation, collagen IV expression, and PKC-*ζ* is not known at present. A well-documented effect of PPAR*γ* is to inhibit TGF-*β* expression and/or TGF-*β* signaling giving rise to an antifibrotic effect, which can be demonstrated in vivo in models of lung fibrosis for example [38, 43]. Given that we have recently found that all of the above effects of high glucose depend on TGF-*β* [44], inhibition of TGF-*β* and its downstream signaling could account for the inhibitory effects of PPAR*γ* observed in the present study. Intriguingly, PPAR*γ* has been variably shown to prevent DNA binding, and in some cases to interact physically with different transcription factors, such as AP-1 [45], Sp1 [33, 46], nuclear factor-1 (NF-1) [47], NF-*κ*B [48]. Therefore, one could hypothesize that suppression of AP-1, Sp1, NF-1, or NF-*κ*B DNA binding could be sufficient to account for the effects of PPAR*γ* by reducing TGF-*β* expression, interfering with the expression of proteins involved in TGF-*β* signaling, and perhaps by directly affecting the expression of some of the above proteins, such as collagen IV, p22^phox^, and VEGF. 

In summary, mesangial cells demonstrate rapid downregulation of PPAR*γ* via both transcriptional and nontranscriptional regulation in response to high glucose. Treatment with rosiglitazone, linked to the PKC-*ζ* pathway, attenuates high glucose-induced ROS generation and prevents VEGF and collagen IV expression through its action on PPAR*γ*. This study suggests that preventing the actions of high glucose on mesangial cell PPAR*γ* may be relevant in the treatment and prevention of diabetic glomerulopathy.
